# Supplementary material for: Spatial transcriptomic analysis identifies epithelium-macrophage crosstalk in endometriotic lesions
Source: iScience. 2025 Jan 10;28(2):111790. doi: 10.1016/j.isci.2025.111790 (PMC11810701; doi:10.1016/j.isci.2025.111790)
Supplement: Document S1. Figures S1–S6 [file mmc1.pdf]

iScience, Volume *28*

Supplemental information

Spatial transcriptomic analysis identifies epithelium-macrophage crosstalk in endometriotic lesions

Gregory W. Burns, Zhen Fu, Erin L. Vegter, Zachary B. Madaj, Erin Greaves, Idhaliz Flores, and Asgerally T. Fazleabas

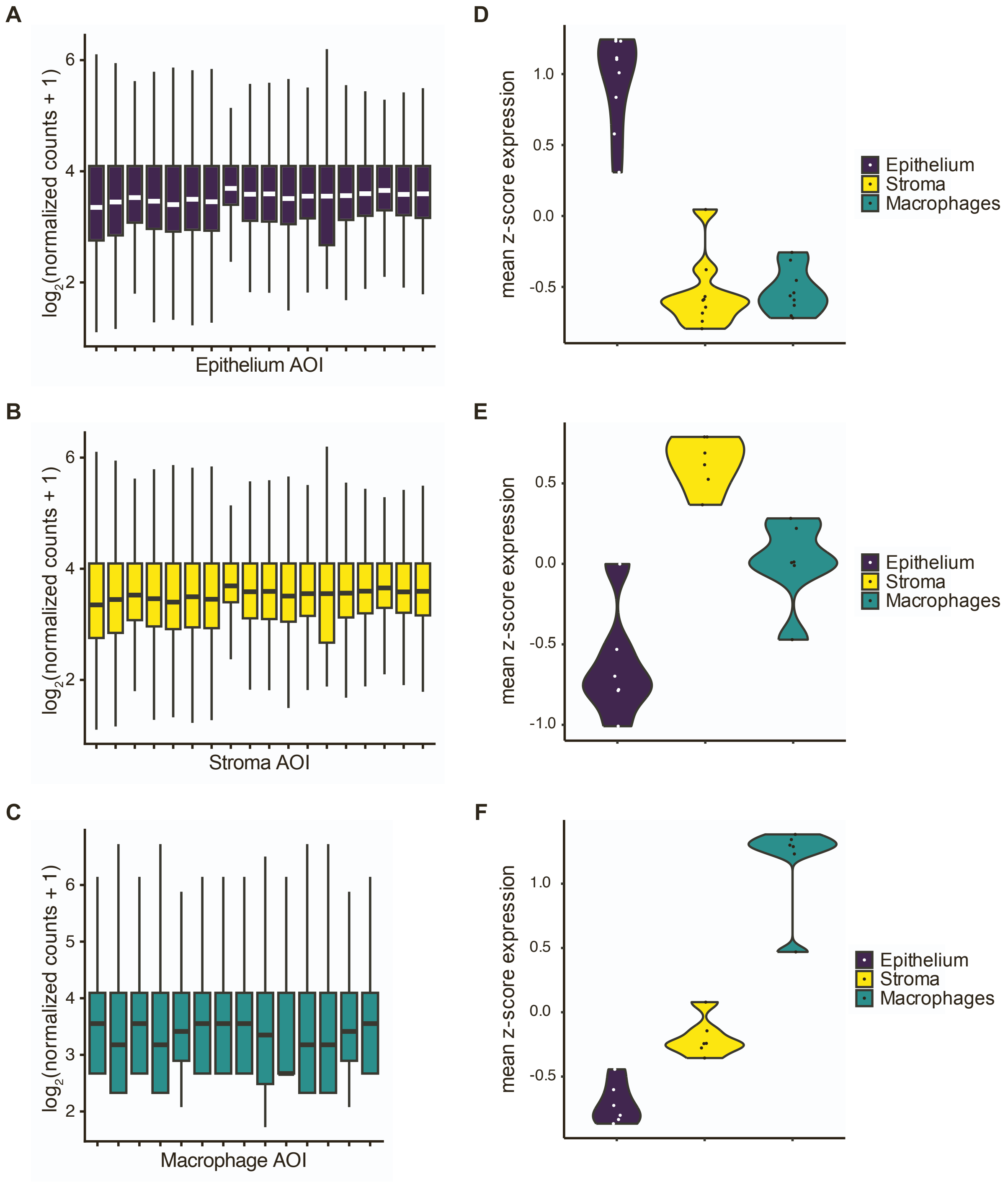

Figure S1

Read count normalization and cell type separation. Normalized read counts for (A) epithelium, (B) stroma, and (C) macrophage segments in box plots, or for areas of interest (AOI) in violin plots.

Canonical marker genes for epithelium (D), stroma (E), and macrophages (F) were compared across cell types to confirm enrichment and cell identities.

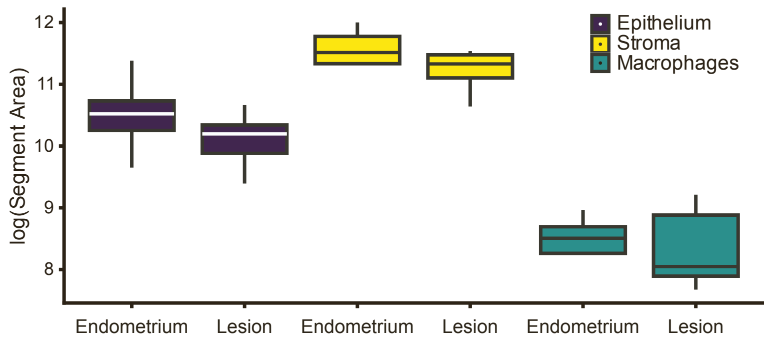

Figure S2

Segment area distributions separated by cell type and tissue shown in box plots.

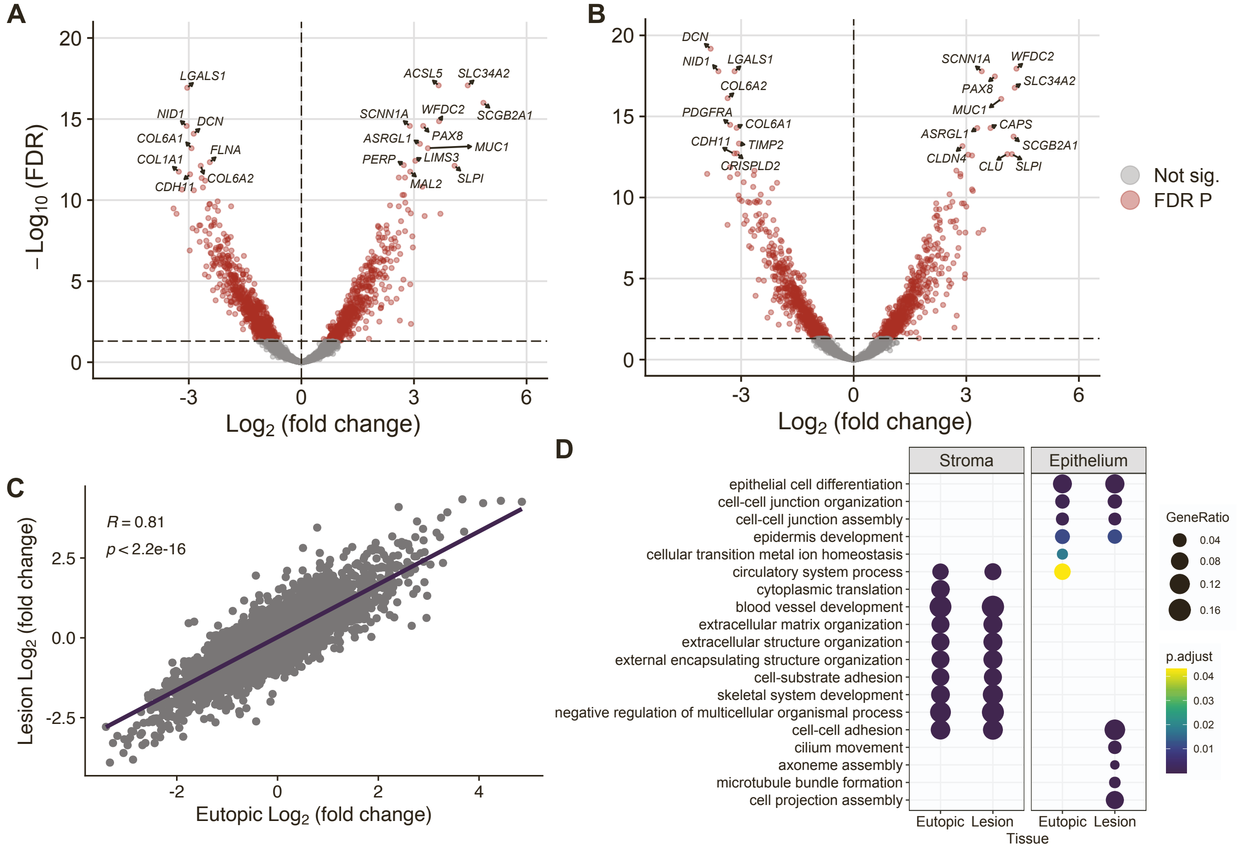

Figure S3

Epithelium versus stroma in eutopic endometrium and lesions. Volcano plots highlighting differentially expressed genes (DEG) in the (A) endometrium and (B) lesion comparisons. There were 1,372 DEG (521 increased and 851 decreased) in the endometrium and 1,313 DEG in lesions (579 increased and 734 decreased). The top DEG were similar in volcano plots with canonical epithelial genes, including *MUC1*, *PAX8*, and *WFDC2*, increased in both epithelia. (C) Cell compartment gene expression was highly correlated in the eutopic endometrium and lesions by fold change. (D) Enriched gene ontology biological process terms were consistent with cell type, including extracellular matrix genes in the stroma and cell-cell junction genes in the epithelium, in the eutopic endometrium and lesions. Several terms related to cell-projections and cilia were unique, however, to the lesion epithelium.

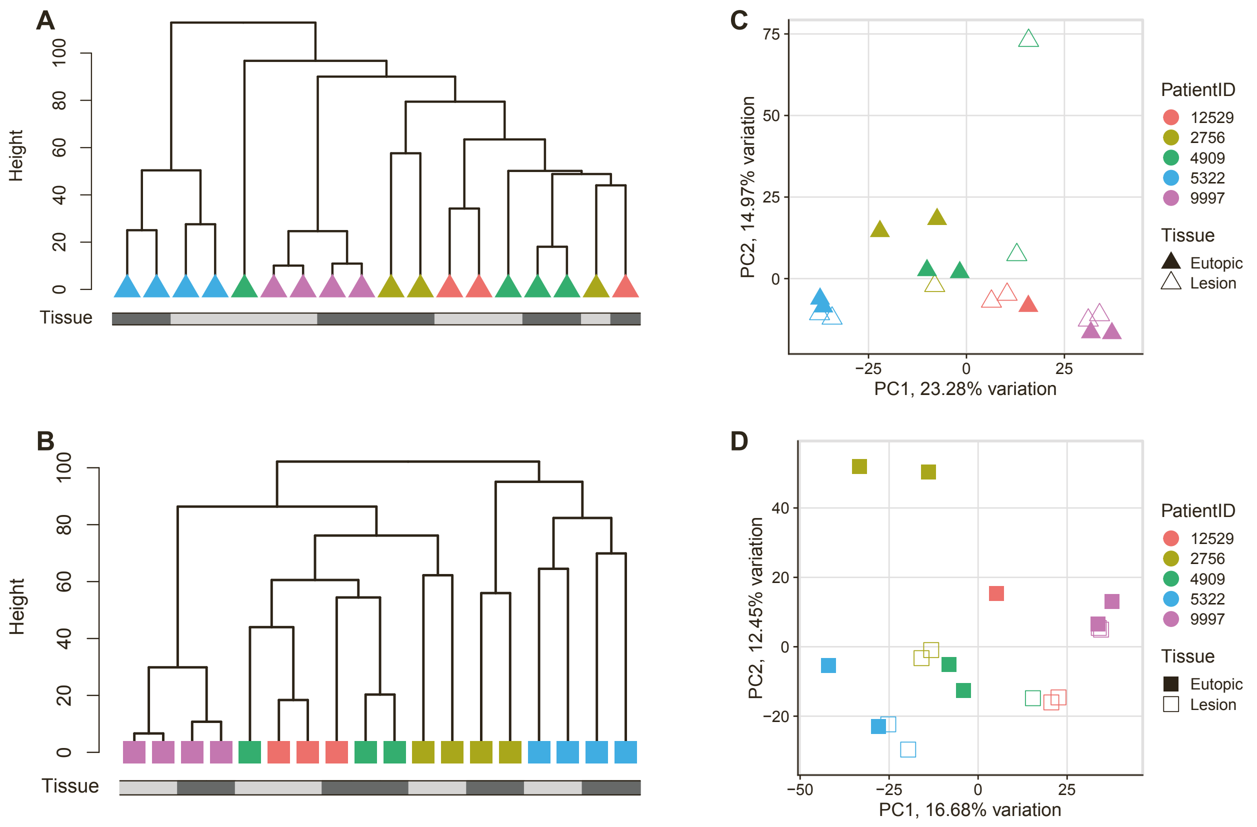

Figure S4

Neither stroma nor epithelium cluster by lesion versus eutopic endometrium. Hierarchical clustering dendrograms of (A) stroma and (B) epithelium segments found no clustering of segments by tissue of origin (endometrium = black bar). Principal component (PC) plots demonstrated that PC1, the largest component of variation, was correlated with patient identity.

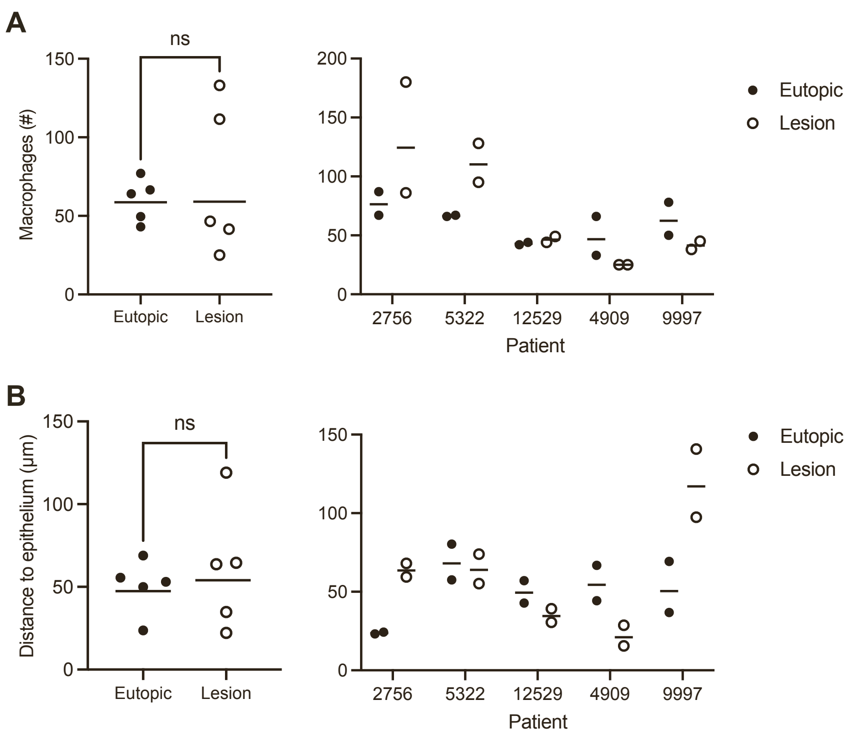

Figure S5

Quantification of macrophages in spatial transcriptomic segments from eutopic endometrium and superficial peritoneal lesions. Data are shown by group and individual patient. (A) Macrophage number was not increased in lesions ($p = 0.98$) and (B) did not appear to be located closer to the lesion epithelium ($p = 0.59$).

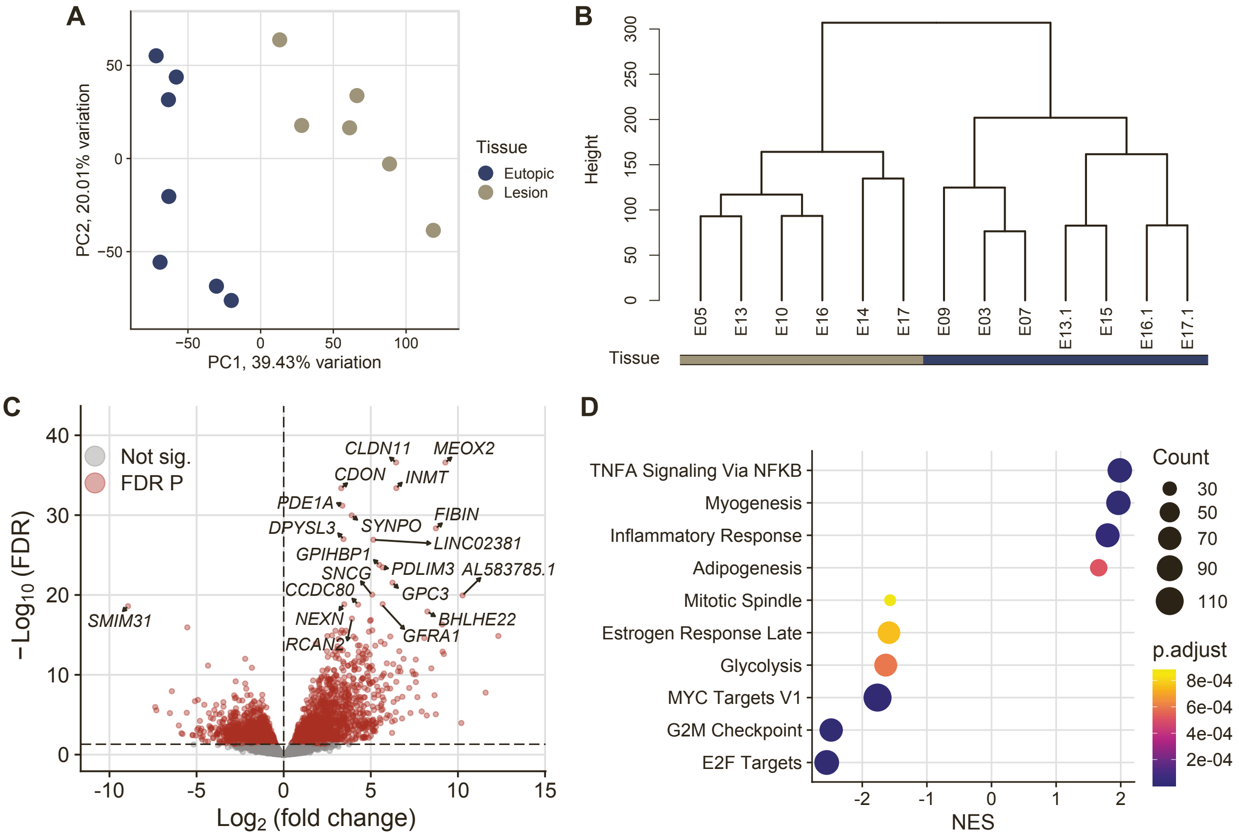

Figure S6

Transcriptome alterations in superficial endometriotic lesions compared to eutopic endometrium as measured by bulk RNA-sequencing. (A) Principal component (PC) plot of superficial peritoneal endometriotic lesions and eutopic endometrium (n = 13) from GSE179640. Samples were separated across PC1 by tissue type, representing 39% of variation in gene expression. (B) Hierarchical clustering dendrogram confirmed separation of lesions from eutopic endometrium. (C) Volcano plot of 3,656 differentially expressed genes in lesions versus endometrium. (D) Gene set enrichment analysis of hallmark pathways found inflammation, represented by TNFα signaling via NF-κB and inflammatory response, increased in lesions while three cell cycle-related gene sets, MYC targets, G2M checkpoint, and E2F targets were decreased. This indicates increased inflammation and decreased proliferation in lesions compared to eutopic endometrium.
